# Process‐Structure‐Formulation Interactions for Enhanced Sodium Ion Battery Development: A Review

**DOI:** 10.1002/cphc.202100860

**Published:** 2022-02-01

**Authors:** M. Anne Sawhney, Malik Wahid, Santanu Muhkerjee, Rebecca Griffin, Alexander Roberts, Satishchandra Ogale, Jenny Baker

**Affiliations:** ^1^ Faculty of Science and Engineering Swansea University Bay Campus, Fabian Way, Crymlyn Burrows Skewen, Swansea SA1 8EN United Kingdom; ^2^ Department of Chemistry Interdisciplinary Division for Renewable Energy and Advanced Materials (iDREAM) NIT Srinagar Srinagar 190006 India; ^3^ Research Institute for Clean Growth and Future Mobility Coventry University Manor House Drive, Friars House Coventry CV1 2TE United Kingdom; ^4^ Indian Institute of Science Education and Research (IISER) Dr Homi Bhabha Road, Pashan Pune 411 008 India; ^5^ Research Institute for Sustainable Energy TCG-CREST Salt Lake Kolkata 700091 India

**Keywords:** cell processing, electrochemistry, electrolyte casting, Na-ion; slurry mixing

## Abstract

Before the viability of a cell formulation can be assessed for implementation in commercial sodium ion batteries, processes applied in cell production should be validated and optimized. This review summarizes the steps performed in constructing sodium ion (Na‐ion) cells at research scale, highlighting parameters and techniques that are likely to impact measured cycling performance. Consistent process‐structure‐performance links have been established for typical lithium‐ion (Li‐ion) cells, which can guide hypotheses to test in Na‐ion cells. Liquid electrolyte viscosity, sequence of mixing electrode slurries, rate of drying electrodes and cycling characteristics of formation were found critical to the reported capacity of laboratory cells. Based on the observed importance of processing to battery performance outcomes, the current focus on novel materials in Na‐ion research should be balanced with deeper investigation into mechanistic changes of cell components during and after production, to better inform future designs of these promising batteries.

## Graphical abstract

Better batteries: This review summarizes the steps performed in constructing sodium ion (Na‐ion) cells at research scale, highlighting variables and techniques that are likely to impact measured cycling performance. Process‐structure‐performance links observed in lithium‐ion cells can be instructive, while parameter optimization through independent validation is recommended when testing novel materials for Na‐ion cells.

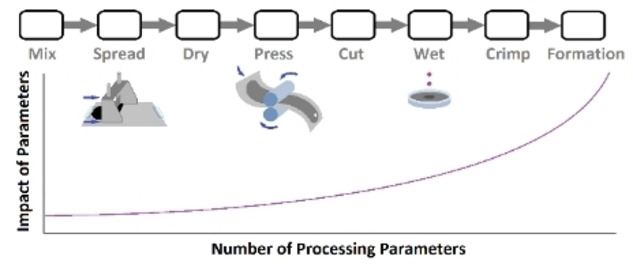

## 1 Introduction

The widespread adoption of renewable energy sources is complicated by inconsistent availability of wind and sun radiation, presenting a need for high volume energy storage before fossil fuel and nuclear generators can be fully replaced.^[1]^ In the current competition to meet the accelerating demand for energy storage technologies, sodium‐ion (Na‐ion) battery development lags that of lithium ion (Li‐ion), Zn‐Air, and redox flow batteries.^[2]^ Na‐ion batteries have several advantages that make them worth pursuing, and they could avoid supply constraints and cost increases as the demand for Li‐ion batteries increases exponentially with the move to electrify vehicle fleets across the world. Alongside cost and supply issues, the use of materials with lower embodied energy and higher abundance than in Li‐ion batteries reduces their environmental impact of manufacture.^[3]^

Na‐ion cells employ the redox potentials occurring between sodium salts and solid electrodes to capture and discharge energy electrochemically. The reversibility of reactions at electrolyte/electrode interfaces is critical for the efficiency and durability of secondary, i. e. rechargeable, Na‐ion batteries. A wide variety of material combinations have been and are continuing to be proposed as Na‐ion cathodes, anodes and electrolyte solvents, but the viability of these choices for a practical Na‐ion battery remains a source of debate among Na‐ion developers.[^4^, ^5^]

Although rechargeable sodium‐based batteries were proposed before the 1970s, the high operating temperatures (∼300 °C) of early designs hindered their application.^[6]^ More recently, unprecedented increases in energy storage requirements – from 10 GWh in 2017 to projections between 181 GWh and 421 GWh in 2030 globally^[7]^ – continue to exceed the supply constraints of any single existing technology. While currently available rechargeable battery systems do offer versatile energy storage solutions, most commercial electrochemical cells contain multiple energy‐intensive, highly flammable and/or toxic materials.^[8]^ An appropriate solution for stationary energy storage would therefore have to prioritise improvements in operational safety and environmental sustainability, combined with scalability at low cost.

Sodium ion battery proponents often highlight widely available and inexpensive materials^[9]^ associated to this type of cell, combined with safety advantages such as stability at zero charge.^[10]^ However, current Na‐ion cell designs employ many of the same hazardous electrolyte solvents, including highly flammable carbonates[^11^, ^12^] and carbon‐intensive compounds such as pyrolyzed anode materials^[13]^ as in the leading industry standard, Li‐ion nickel manganese cobalt oxide (NMC) batteries. Na‐ion cell materials are also often assumed to have inherent cost advantages due to the natural abundance of sodium compounds, but this economic benefit has yet to be realized.^[14]^ Although Na‐ion cells have been demonstrated in prototype systems including transport applications, commercially available examples still underperform in energy density when compared to Li‐ion cells; a list of commercially produced cells are given in Table 1.

**Table 1:** Representative Na‐ion and Li‐ion commercial cells at time of writing with declared capacity.

Type	Company	Cathode	Anode	Energy Density [Wh/kg]	Ref.
*Na‐ion*	CATL	prussian white (sodiated prussian blue analogue)	hard carbon	160	[15]
	Faradion	layered nickelate; Na_a_Ni_(1‐x‐y‐z)_Mn_x_Mg_y_Ti_z_O_2_	hard carbon	140	[16]
	HiNa	layered oxide (unspecified)	soft carbon	145	[17]
	Natron	NaFe[(Fe(CN)_6_] (prussian blue)	prussian blue	50	[18]
	Novasis	prussian blue analogue	hard carbon	100–130	[16, 19]
	Tiamat Energy	Na_3_V_2_(PO_4_)_2_F_3_	hard carbon	122	[19]
					
*Li‐ion*	LG Chem	lithium nickel, cobalt manganese (NMC)**	SiOx−C**	202*	[20**, 21*]
	CATL	NMC	graphite	215	[22]
	BYD	lithium Iron Phosphate (LFP)^†^	graphite	140–170^†^	[23^†^, 24]
	Panasonic	lithium Nickel‐Cobalt‐Aluminum Oxide (NCA)^≠^	graphite^≠^	240	[25^≠^, 26]
	Tesla	NCA	Si‐C	300	[23]

One reason often identified to explain low energy density in Na‐ion cells is the higher atomic weight of sodium relative to lithium,^[27]^ but these elements represent less than 1 % of total cell volume.^[28]^ In contrast, the mass of current collector foil contributes substantially to the total cell weight;^[29]^ this favours Na‐ion anodes, which unlike Li‐ion anodes, can employ less dense aluminium rather than copper as a current collector. Combined with higher copper demand for renewable energy installations, this substitution is a further driver for Na‐ion compared with Li‐ion.^[30]^ Na‐ion cell design often employs similar precursor materials to those in Li‐ion cells, including redox‐active salts of hexafluorophosphate (PF_6_), the cost of which has increased sharply with the growth of Li‐ion production.^[31]^ In contrast, higher demand for sodium salt is unlikely to cause cost volatility due to broad geographical distribution and multiple extraction methods for these compounds.^[32]^ The cross‐platform translation of electrolyte composition persists despite consistently observed differences in electrochemical phenomena between these two cell types^[33]^ such as unfavourable sodium‐graphene interactions reducing selected carbonate solvent molecules.^[34]^

Conventional procedures employed in fabricating Li‐ion cells are also frequently adopted for Na‐ion development.^[35]^ This mimicking of practices is sometimes justified by the convenience of employing existing Li‐ion battery manufacturing equipment seamlessly for Na‐ion production.^[36]^ However, the efficiency of this industrial equivalency cannot be assured without extensive testing and optimization of Na‐ion fabrication methods suited to each battery concept and architecture. Since sodium‐based cathode materials are more reactive to humidity than Li‐ion equivalents, standard dry‐room conditions used in Li‐ion cell assembly may not be appropriate for Na‐ion production,^[36]^ and formation cycles used to complete Li‐ion cells yield very different results with Na‐ion.^[36]^ For example, both alkylcarbonate solvents and perchlorate salts considered stable in Li‐ion electrolyte were found to decompose in Na‐ion cells, limiting both capacity and cycle life.^[37]^ Testing and quality control methods used on Li‐ion electrodes also may not be appropriate for Na‐ion counterparts, since the latter are more likely to alloy with contaminant metals or form dendrites.^[37]^

Decades of process improvement favours Li‐ion as a more mature technology. Some process‐structure‐performance relationships have been found to apply widely to Li‐ion cells,^[38]^ most notably the effect of drying rate on electrode adhesion to current collector.^[39]^ These observed process‐based effects could be instructive in guiding Na‐ion procedures, provided systematic testing is performed across a range of typical Na‐ion cell material combinations. This review aims to summarize these critical cell production variables, identify factors in experimental methods particularly critical for optimizing performance of new Na‐ion materials, and encourage researchers to scrutinize procedures adopted from Li‐ion practice before assuming equivalence for Na‐ion systems.

### 1.1 Na‐ion Cell Conventions

Contemporary Na‐ion cells often consist of a layered metal oxide cathode, intercalation‐type carbon anode and electrolyte composed of fluorinated salt in carbonate ester solvent, though the reversible capacities of this combination effectively limits energy density near 150 Wh/kg.^[40]^ Some proposed Na‐ion designs favour non‐standard materials such as aqueous electrolyte with advantages in safety, cost or resource availability but compromising energy density, such as 40 Wh/kg reported in a symmetrical cell employing sodium sulphate based aqueous electrolyte.^[41]^ Nonetheless, increased energy density is frequently targeted in Na‐ion research, which is quantified with test methods directly adopted from Li‐ion development.

Theoretical cathode capacities are lower than those of anode materials, but operationally, cell performance is limited by the passivating layer at the anode interface, called the solid‐electrolyte interphase (SEI).^[42]^ Compared to Li‐ion anodes, the SEI on Na‐ion anodes is less stable, motivating continued investigation into alternative chemistries such as diglyme based electrolytes to create more durable and uniform SEIs than those occurring in common alkyl carbonate solvents.^[43]^ Additives have frequently been used to stabilise the SEI subsequently improving cycle life, with tris(trimethylsilyl) phosphite (TTSPI) and vinylene carbonate (VC) shown to give improved capacity retention when compared to the Li‐ion standard, fluoroethylene carbonate (FEC).^[44]^ Hard carbon remains the standard choice for Na‐ion anodes,^[45]^ though alternative materials might offer improvements to irreversible capacity loss and SEI solubility; this includes metal alloys as active electrode material or ether substitutes to typical alkyl carbonate electrolyte solvents.^[46]^

Cell assembly and testing techniques also vary between laboratories, preventing direct comparison between electrode materials highlighted in different studies, since procedural variables in experimental methods such as mixing procedures substantially impact measured capacity.^[47]^ Inappropriately selected parameters at any step of electrode fabrication would be expected to result in divergence from theoretical capacity,^[48]^ while underexplored properties of electrolyte such as viscosity and ionic compatibility require further study to understand their effect on electrochemical stability.^[49]^ The majority of current research in Na‐ion cell design aims to improve capacities through proposing novel materials and chemical additives, while the opportunities of adjusting cell fabrication processes is sparsely reported.^[50]^

### 1.2 Na‐ion Cell Fabrication

**Figure 1:**
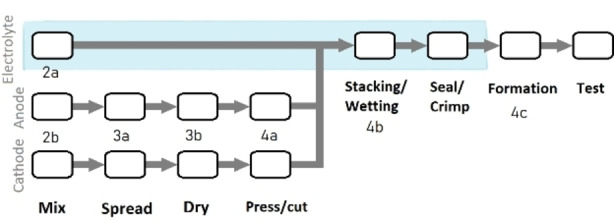
Steps of cell production at research scale indicating the corresponding sections in this text; blue shading indicates steps within inert (dry room or argon) atmosphere.

The steps of cell production at both large and small scale begins with mixing raw materials (Figure 1). Although the active material, binder and solvent used in cathodes can be very different from those applied in anodes, the production processes to make these films are very similar^[51]^ and undertaken in the lab environment rather than a dry room or glove box. In contrast, electrolyte components are generally handled entirely within an inert environment due to their inherent volatility and reactivity. Once dry electrodes have been cut into a shape suited for cell dimensions, they may be stacked into place before the cell is filled with electrolyte, but these two steps are often combined in a research setting^[52]^ where coin cells can be assembled within the restrictive space of an argon glovebox.

Even for initial mixing of the electrode slurry, a wide range of equipment and parameters could be applied. For manually performed activities, the duration of a processing step is often judged by subjective criteria, such as the visual appearance of a smooth mixture. Variability between laboratory facilities and between production batches compromises the repeatability of results, which has motivated some Li‐ion researchers to publish evidence‐based recommendations on best practice.[^52^, ^53^]

By publishing photographs of equipment and custom testing hardware, the same techniques applied by Marks et al.^[53]^ could be easily reproduced by other Li‐ion cathode researchers. The relatively simple methods proposed by the authors effectively demonstrated the effect of formula and processing variables on electrode adhesion to current collector foil, which is often a performance‐limiting factor.

In a later publication, members of the same group demonstrated the importance of electrode alignment and appropriate selection of separator type for Li‐ion coin cells.^[52]^ Although these technique details apply only to coin cell research, this is common for investigations of novel cell materials, which could benefit from the improvement in capacity retention observed when the recommended practices were applied. These approaches could also be instructional to the Na‐ion research community, though they cannot replace validation and optimisation of processing methodology specific to each facility and cell chemistry. In particular, standard electrolyte solvents used in both Li‐ion and Na‐ion cells often require heating and pre‐mixing before adding a salt, which often does not dissolve or form a stable suspension.[^54^, ^55^] Techniques in electrolyte mixing therefore affect the accuracy of salt concentration in electrolyte, which determines the availability of the active ion; a principal factor in cell performance. This subject of mixing may be the least discussed of all cell production processes, therefore it is the first topic addressed in this review.

## 2 Combining and Mixing

The first step in construction of a sodium‐ion battery generally consists of mixing powders into solvent, which applies to both electrode and electrolyte fabrication. Raw materials, as electrode/electrolyte formula ingredients or their precursors, can usually be procured from commercial chemical suppliers,^[56]^ minimizing inter‐batch variability and impurities. In contrast, the techniques used to blend these materials together may vary greatly between experimenters. For example, the homogeneity and process‐ability of electrode slurries depends on interactions of solid particles suspended in a binder solution, introducing a range of additional variables for optimization. This section summarizes the most common mixing methods described in sodium‐ion research while highlighting the advantages and disadvantages of these techniques based on evidence from parallel fields.

### 2.1 Electrolyte

At the time of writing, most Na‐ion cells in research and commercial applications employ liquid electrolyte, composed of a redox active sodium salt dissolved into a blend of solvents. By percolating into the pores of solid electrodes, or wetting, fluid electrolyte increases the active surface area of the electrolyte/electrode interface. In contrast, developments in gel polymer or ceramic electrolyte could offer advantages in safety, manufacturing simplicity and practicality. There are 3 main types of solid‐state electrolytes (SSEs) for Na‐ and Li‐ion batteries: solid inorganic/ceramic electrolytes (crystalline or glasses), organic polymer electrolytes and hybrid solid electrolytes, which mix inorganic SSEs with polymers or liquid electrolytes.

#### 2.1.1 Liquid Electrolytes

The choice of mixing strategies in the case of liquid electrolytes is not much discussed owing to the easy miscibility among the polar solvents. The dissolution of salt in the polar solvents is similarly self‐driven and hence is little affected by the type of mixing method. However, cell performance has been seen to be sensitive to the composition of electrolyte systems, and compositional engineering has been extensively explored for performance enhancement.

The most common liquid electrolyte systems employed in the Na‐ion battery include the salts NaPF_6_, NaClO_4_, NaFSI, NaTFSI, and NaFTFSI,^[57]^ in carbonate (ether and ester) or ionic liquid solvents.^[58]^ Though the design of Na‐ion batteries has thrived on the general understanding of Li‐ion batteries, the choice of electrolyte for the former is still being optimized. The solvent viscosity, polarity, and structure impose a direct influence on the electrochemical performance by controlling the underlying ion‐ion and ion‐solvent interactions.

The carbonate esters are the most common electrolyte solvents for Na‐ion. Primarily, propylene carbonate (PC) has received consideration due to higher dielectric constant (64.9 at 25 °C) and broad effective temperature range (melting point −49.2 °C and boiling point 241.7 °C).^[59]^ Though highest in terms of polarity with a dielectric constant of 89.8, ethylene carbonate (EC) is solid at room temperature and therefore is not used individually; occasionally a 1 : 1 mixture of EC and PC, dimethyl carbonate (DMC) or diethyl carbonate (DEC) is employed reducing the viscosity to <3.2 mPa.s at 20 °C.

Viscosity is critical, as it influences the solvation kinetics of electrolyte and hence in turn exercises control on the cation/anion availability at the sites of redox activity. Viscosity can be measured using a standard cone and plate set up, which will determine viscosity at different strain rates and so determine if the electrolyte (or slurry) is Newtonian in behavior or if it exhibits non‐Newtonian behavior such as shear thinning.

Particularly in the case of non‐aqueous Na‐ion batteries, where carbonate solvent mixtures are employed, the viscosity holds a pivotal role in deciding the electrochemical performance. Che et al.^[60]^ revealed that electrolyte viscosity and thermo‐chemical stability directly intervenes in the cathode interfacial structure and composition in the case of NaPF_6_ dissolved in carbonate solvents including ethyl methyl carbonate (EMC), DMC or DEC with PC or EC. The authors concluded that PC/EMC electrolyte, with intermediate viscosity and conductivity (of 2.6 mPa.s and 7.3 mS cm^−1^ respectively), yields the best stability compared to other carbonate systems.^[60]^ In practical applications within the lab, co‐solvents are required to lower the viscosity of electrolytes containing EC, which is solid at room temperature. Crystalline at room temperature, EC is usually heated above melting point (36.4 °C^[60]^) in order to mix with a lower viscosity co‐solvent.

Ether‐based electrolytes have attracted attention due to successful sodium intercalation in graphite with these solvents. In contrast to the carbonate solvents, sodium salts play a more passive role in determining intercalation capacity and rate performance.^[61]^ This has been expanded by the work of Morales et al.,^[62]^ who observed limited solubility of LiPF_6_ in glymes (saturating above 0.5 M) compared to NaPF_6_ (temperature‐stable at 0.8 M), revealing the ion‐solvent association and solvent chain length also affect achievable ranges of electrolyte ionic conductivity (from <10^−3^ to >10^−2^ S cm^−1^).

A separate category of liquid electrolyte, called ionic liquid (IL), has been proposed as a less flammable option with minimal compromise to ionic conductivity (10 mS cm^−1^ at room temperature,^[63]^). This type of electrolyte consists of a sodium salt or blend of salts that remains liquid at room temperature. Only a few out of a diverse variety of possible salts have been proposed as an IL electrolyte for Na‐ion batteries, typically TFSI^[63]^ or other fluorine‐containing anion with a pyrrolidinium or imidazolium cation.^[64]^ Based on equivalent processes for Li‐ion cell research, IL salts are mixed using procedures typical for carbonate‐based liquid electrolytes,^[65]^ sometimes preceded by a purification step such as heating under vacuum.^[63]^ IL electrolytes tend to be more viscous than typical liquid electrolytes,^[65]^ and they may also be applied in combination with carbonate electrolyte solvents^[66]^ or even integrated with a solid polymer gel electrolyte membrane.[^66^, ^67^]

#### 2.1.2 Polymer Electrolytes

The assembly of polymer electrolytes involves the union of the electrolyte part and the separator part of traditional liquid electrolytes. However, it demands inherent structural modifications and compositional engineering towards achieving comparable ionic conductivities with solid‐like mechanical robustness, thermal stability, and flexibility. In quantifying performance, ionic conductivity of 10^−5^–10^−3^ mS cm^−1^ is typical^[68]^ compared with 10–15 mS cm^−1^ for liquid electrolytes. The common components of a solid polymer electrolyte include the polymer matrix, organic solvents as plasticizers, electrolyte salts immobilized on the polymer matrix and inorganic particles as fillers. The solid nature of the electrolyte implies that performance will be influenced by the fabrication process, as with electrode components.

**Figure 2:**
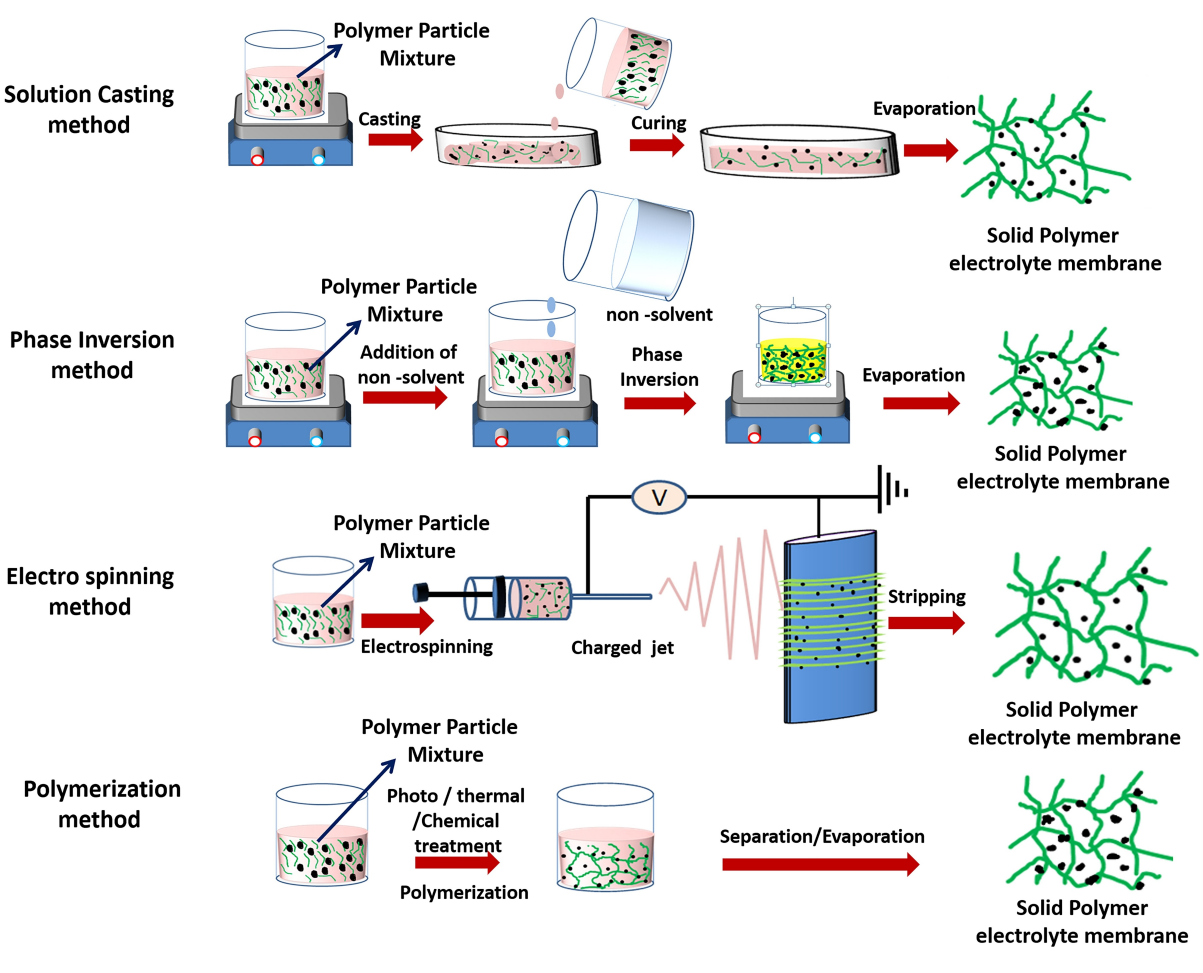
Protocols for polymer electrolyte membrane fabrication.

Pore engineering is an essential component of the electrolyte fabrication process as it directly relates to ionic conductivity, with fine pores in poly(vinylidene fluoride‐ co ‐hexafluoropropylene) (PVdF‐HFP) based electrolytes enabling greater amounts of entrapped liquid electrolyte prior to the formation of the gel‐like polymer electrolyte.^[69]^ The other important external component is the fillers that help improve the mechanical strength (from 3.1 MPa tensile strength to 9.86 MPa,^[70]^) and ionic conductivity (from 0.22 mS cm^−1^ to 0.68 mS cm^−1^,^[71]^). Various processing strategies have been implemented with polymer electrolyte assemblies: solution casting,^[66]^ phase separation,^[67]^ electro‐spinning^[68]^ and in‐situ polymerization,^[72]^ shown in Figure 2.

The solution casting technique involves dispersion of electrolyte components in a solvent followed by casting into a container of desired geometry and drying through evaporation. For example, Zhang et al.^[73]^ mixed poly(ethylene oxide) with NaPF_6_ in acetonitrile, which was subsequently evaporated by drying for two days, firstly at room temperature and then under a vacuum. Xue & Quesnel^[74]^ used the solution casting strategy to combine poly(methyl methacrylate) (PMMA) and polycarbonate electrolyte with NaBF_4_ then slowed solvent evaporation with a glass cover to prevent surface film formation. Mishra et al.^[75]^ added Al_2_O_3_ nanoparticles during solution casting of polymer electrolyte to improve ionic conductivity from 7.5×10^−4^ S cm^−1^ to 1.5× 10^−3^ S cm^−1^, attributed to the increased dissociation of cation‐anion pairs. However, the cell capacity rapidly faded with cycling, which was attributed to loss of ionic mobility from hardening of the gel.

Chemical surface modification for incorporating flexibility in the polymer electrolyte, has been applied to optimize the solution casting technique. In this regard, Gao et al.^[76]^ reported composite gel–polymer/glass–fiber electrolyte with PVDF‐HFP reinforced by a glass–fiber paper and modified by a polydopamine coating, applying a modified solution casting method, giving mechanical strength and ionic conductivity of 5.4 mS cm^−1^.

The phase separation technique involves dispersing a polymer component and inorganic component in a binary mixture of solvent and non‐solvent, causing the polymer to coat the inorganic particles, followed by drying through evaporation. For example, a PVdF‐HFP membrane was prepared by phase separation^[77]^ using water as the non‐solvent to tune pore structure in the polymer. Kim et al.^[78]^ applied water with acetone as the non‐solvent for pore control in a phase separation fabrication of PVdF‐HFP, reporting considerably higher ionic conductivity at 3.8 mS cm^−1^.

Phase inversion is a special case of the phase separation technique involving three steps: dispersion in a mixture of a solvent and non‐solvent, coating on a substrate, and drying for sequential removal of solvent and non‐solvent to obtain a porous electrolyte film. Phase inversion techniques can impart high porosity of over 70 % to polymer membranes. Verma, Mishra & Rai^[79]^ applied phase inversion to produce PVdF‐HFP membranes using dimethyl formamide (DMF) as solvent and TiO_2_ nanoparticles for pore tuning, but differences in sodium salt and electrode composition undermine direct comparisons of reported ionic conductivity with other studies.

In situ polymerization methods aim to simplify processing by applying fewer steps and/or reagents than solution casting or phase separation. A recent study applying uncomplicated synthesis of NaPF_6_ into cross‐linked 1,3‐dioxolane and trimethylolpropane triglycidyl reported high stability at room temperature, with corresponding ionic conductivity of 0.82 mS cm^−1^.^[80]^ Two years earlier, Zheng et al.^[81]^ achieved a much higher ionic conductivity (6.29 mS cm^−1^) at room temperature with an in‐situ polymerized gel combining three polymers, though this followed several synthesis steps to produce a novel precursor. Bella et al.^[82]^ described the inherent processing complexity of producing free‐standing gel electrolytes, and proposed photo polymerization through UV curing as a simpler alternative.

In contrast, the electrospinning process involves the dispersion of polymer in co‐solvents before nozzle injection into a high‐voltage electric field through a rotating drum to achieve fiber mats, filler impregnation then stripping the membrane, often followed by soaking in liquid organic electrolyte. A recent example of this approach exploited properties of three different polymers (poly(siloxane‐g‐ethylene oxide, polymethylhydrosiloxane and methoxypolyethylene glycols) in a multi‐step two‐solvent process to produce flexible electrolyte membranes that demonstrated over 86 % capacity retention after 1000 cycles in a Na‐ion cell.^[83]^ Similar ionic conductivities were achieved with fewer reagents by Freitag et al.,^[84]^ who electrospun poly(ethylene oxide) (PEO) with and without succinonitrile (SN), a plasticizer used for electrospinning Li‐ion polymer electrolytes. Freitag et al. revealed fundamental differences in ionic mobility through these membranes between sodium and lithium, with SN giving no significant improvement in the conductivity in the sodium‐based system at room temperature unlike the lithium system. The authors explained these differences by contrasting associations of sodium and lithium ions with the polymer chains and plasticizer, advising against applying the same formula to both cell types.^[84]^

Janakiraman et al.^[85]^ electrospun PVDF to produce a separator subsequently soaked in NaPF_6_ to form a gel, yielding an ionic conductivity of 1.08 mS cm^−1^ while using materials already common to Na‐ion cell production. The authors attribute the higher conductivity obtained with sodium salt, compared with identical tests using LiPF_6_ (which yielded an ionic conductivity of 0.94 mS cm^−1^), to ionic charge density and weaker interactions of the sodium with the fluoride ion.^[85]^ However, the solvents used (equal parts EC and PC) are not conventionally applied in Li‐ion electrolyte, presenting another complication to direct comparisons between Na‐ion and Li‐ion systems. A porous membrane structure (78 %,^[85]^) with low crystallinity was generally associated with higher ionic conductivity in polymer electrolytes.

These examples of novel polymer electrolytes are summarized in Table 2. The focus of polymer electrolyte studies on chemical characterization, including sodium transference and ionic conductivity, contrasts with relatively little discussion of mechanical resilience in these membranes. Despite frequently mentioned benefits of mechanical properties such as elasticity and strength, these are rarely tested or quantified in polymer electrolyte assessment. A few exceptions suggest opportunity exists for repeatable, uncomplicated mechanical testing of gel membranes, such as in Yang et al..^[77]^ By applying an industry‐standard protocol using a tensile tester machine on samples of defined size, the authors compared stress vs. strain curves between pieces of separator and the proposed polymer electrolyte.^[77]^ A simpler test was performed by Bella et al.,^[82]^ in which polymer films were bent 50 times around a 2.5 mm rod to confirm flexibility through visual inspection. Recently, an abbreviated form of both these methods was applied by Li et al.,^[83]^ though the ambiguity of equipment used in stress‐strain testing and degree of folding in visual bend tests prevents replication of these techniques, which could otherwise be adopted as useful standards for mechanical evaluation. The choice of fabrication method is critical in the context of achievable flexibility, porosity and conductivity for a given set of polymer, filler, and plasticizer composition.^[68]^

**Table 2:** Sample polymer electrolytes reported in literature, listed by fabrication process.

Fabrication Method	Composition	Reported ionic conductivity [mS cm^−1^]	Test conditions	Ref.
solution casting	Poly(ethylene oxide) (PEO)+NaPF_6_	0.63	Ionic conductivity tested by EIS from 20 °C to 80 °C between steel electrodes, reported value at 80 °C	[73]
	PVdF–HFP/PMMA+Al_2_O_3_+NaCF_3_SO_3_ EC/PC	1.5	Ionic conductivity tested by EIS from −50 °C to 100 °C between steel electrodes in nitrogen environment, reported peak at 70 °C	[75]
	PMMA+Polycarbonate+NaBF_4_ EC/PC	0.57	Ionic conductivity tested by EIS from 20 °C to 90 °C between aluminum electrodes, reported value at “room temperature”	[74]
phase separation	PVdF–HFP+NaClO_4_ EC/DMC/DEC	0.6	Ionic conductivity tested by EIS from 25 °C to 75 °C between steel electrodes, reported value at “ambient temperature”	[77]
	PVDF‐HFP+glass fiber+NaClO_4_ EC/PC	3.8	Ionic conductivity tested by EIS between steel electrodes, reported value at 25 °C	[78]
	PVdF‐HFP+TiO_2_+NaPF_6_ EC/PC	1.3	Ionic conductivity tested from EIS from 30 °C to 80 °C between steel electrodes, reported value at “room temperature”	[79]
chemical cross‐linking/ polymerization	1,3‐dioxolane, trimethylolpropane triglycidyl+NaPF_6_ PC/FEC	0.82	Ionic conductivity tested by EIS from 20 °C to 70 °C in sodium metal coin cells, reported value at “room temperature”	[80]
	methyl methacrylate and trifluoromethyl methacrylate+phosphonate cross‐linking agent+NaClO_4_ EC/PC/FEC	6.29	Ionic conductivity tested by EIS at 25 °C and 60 °C between steel electrodes, reported value at “room temperature”	[81]
	bisphenol A ethoxylate dimethacrylate+poly(ethylene glycol) methyl ether methacrylate+TiO_2_+NaClO_4_ PC	5.1	Ionic conductivity tested by EIS from −10 °C to 80 °C between steel electrodes, reported value at 20 °C	[82]
electro‐ spinning	polymethylhydrosiloxane+methoxypolyethylene glycols+Polyacrylonitrile+NaClO_4_	1.06	Ionic conductivity tested by EIS from 25 °C to 85 °C between steel electrodes, reported value at “room temperature”	[83]
	PEO+succinonitrile+NaBF_4_	1.00	Ionic conductivity tested by EIS from 293 K (19.85 °C) to 328 K (54.85 °C) between steel electrodes, reported value at 54.85 °C	[84]
	PVDF+NaPF_6_ EC/PC	1.08	Ionic conductivity tested by EIS from 26 °C to 75 °C between steel electrodes, reported value at “ambient temperature”	[85]

PEO=Poly(ethylene oxide), PVDF=Poly(vinylidenedifluoride), PVDF‐HFP=Poly(vinylidenedifluoride‐co‐hexafluoropropylene), PMMA=Poly(methyl methacrylate).

#### 2.1.3 Ceramic‐Based Solid Electrolytes

**Figure 3:**
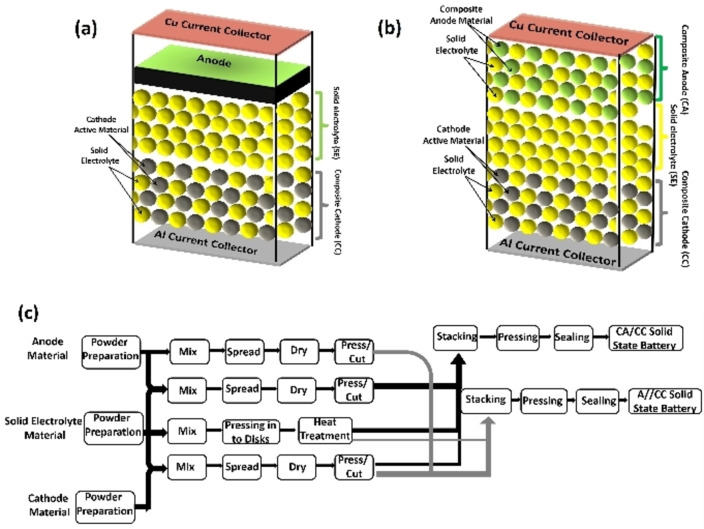
Schematic for solid‐state battery architecture: a) Normal anode–composite cathode architecture, b) Composite anode–composite cathode architecture, c) Schematic for fabrication of composite anode/composite–cathode (CA/CC) and anode/composite cathode (A//CC) inorganic solid‐state batteries.

Solid electrolyte architecture differs considerably from gel and liquid electrolyte Na‐ion cells in that the electrode components have a uniform composition consisting of solid electrolyte and electrode active materials. Figures 3a and 3b show the typical architectures of interface optimized solid‐state Na ion cells. For the preparation of composite electrode materials with intimate electrolyte contacts, solid electrolyte and cathode/anode slurries are mixed separately, followed by pressing the composite into a disk and subsequent heat treatment (see Figure 3c).

Many aspects of Na‐ion batteries originate from Li‐ion battery manufacturing techniques; however, Na solid‐state electrolytes (SSEs) have a longer history of research in the field of solid‐state batteries. Even before the creation of Li‐ion batteries, Goodenough et al. (1976) synthesised a Na super‐ionic conductor (NASICON).^[86]^ The common formula of NASICON is Na_1+x_Zr_2_P_3‐x_Si_x_O_12_, an inorganic ceramic with a crystal 3D framework which enables high ionic conductivity.

NASICON is widely reported in literature, however, it has several issues that reduce its ionic conductivity: secondary phase formations, charge transfer resistance/interface issues and difficulties in manufacturing. Microstructure (electrolyte porosity and grain size) is shown to impact the electrolytes performance/conductivity and is affected by preparation/synthesis methods. NASICON is synthesised by two main routes: solid‐state reaction and sol‐gel synthesis.^[87]^ There are numerous manufacturing processes that affect the outcome of the solid state reaction, which are related to the size and break‐up of the particles, subsequent mixing and the formation of pellets which can be sintered, where time and temperature will differ dependent on article size and pellet geometry.^[88]^

Sol‐gel is a more complex technique compared to solid‐state reactions, however both techniques require refining as they do not commonly produce monoclinic phases.^[89]^ As an example, the stoichiometry of NASICON is often modified to alter the electrochemical performance of the electrolyte. Park et al.^[90]^ used an excess of sodium by increasing the ratio of the sodium precursor, which in turn changed the stoichiometry of NASICON. Characterisation indicated an alteration in the grain structure of the electrolyte and an improvement in the total ionic conductivity.^[90]^ Alternatively, the chemical precursors for both solid state and sol‐gel synthesis are often substituted to achieve the same effect. Rao&Patro^[91]^ created an excess of Na by testing two different Na precursors, Na_2_CO_3_ and Na_3_PO_4_, both of which also increased ionic conductivity compared to its standard stoichiometric equivalents.

Another example is the variation of deposition methods for sol‐gel synthesis. Shimizu&Ushijima^[92]^ successfully used spin coating to make thin film gels, in contrast to Martucci et al.^[93]^ who developed multilayer films via dip coating. Sintering parameters and methods are also variable; demonstrated by Narayanan et al.^[94]^ who explored the trade‐off between sintering durations and temperatures. The work suggested that the sintering parameters are a factor in determining the conductivity and density of NASICON, as temperature can influence the volatisation of components.^[94]^

There are several attempts at synthesising the Li equivalent of NASICON, the first attempt was reported by Hong^[95]^ called LISICON. The structure has a similar framework, LiZr_2_(PO_4_)_3_, however the electrolyte had a lower ionic conductivity than the Na equivalent.^[96]^ Substituting Zr with other cations (e. g. Ge, Ti, Hf) can increase conductivity values, and therefore various variations on this structure have been reported since; for example, LATP, and LAGP.

Across most solid‐state electrolyte manufacturing for both NASICON and other types, two main mixing methods have been recognized: mortar and ball milling, both further including wet and dry methods. For example, Deng et al.^[97]^ ball milled dry electrolyte precursors into a powder in one step before pressing into a pellet, while Lan et al.^[98]^ ball milled precursor powder in ethanol as only one of multiple wet steps. The latter design aimed to optimize the cathode/electrolyte interface, since interfacial resistance between the solid electrolyte and cathode is a known performance limitation of SSE Na‐ion batteries.^[98]^

Though promising for next‐generation energy storage technology, solid‐state batteries are currently limited by insufficient electronic and ionic efficiency. Additionally, mechanically dynamic solid electrolyte/electrode interfaces present unique challenges for design of viable solid‐state cells.^[99]^ The particle size, porosity, and thickness of the electrode films also have influence on the overall performance, as explained in the following section.

### 2.2 Electrode Slurries

Slurry characteristics such as viscosity, surface tension and separation stability are directly related to micro and nano‐level particle‐particle interactions.^[100]^ These interactions can be manipulated through the application of fluid shear forces, heat, co‐solvents or post‐agitation resting. Consequently, the temperature, time, speed, apparatus type and even the order of adding materials during mixing all impact particle size distribution, homogeneity and shear resistance of a slurry. Evidence from lithium‐ion experimentation demonstrates these metrics also impact the electrochemical characteristics of electrodes after drying.[^101^, ^102^]

**Figure 4:**
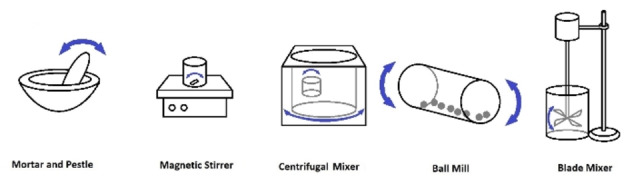
Devices commonly applied for slurry mixing at laboratory scale.

The convention of quantifying electrode composition is by dry weight, rather than as a wet slurry. While material proportion is an important specification, variability in mixing particle suspensions and binder solutions can lead to different responses from electrodes of identical chemical content.^[103]^ Inter‐electrode imprecision can be expected from mixing with different speeds or durations, such as hand‐grinding or magnetic stirring until visual homogeneity is subjectively determined. Alternatively, several laboratory‐scale devices can be employed on small slurry volumes to standardize these parameters (see Figure 4).

One of the most frequently used wet slurry blending devices in electrode research is a centrifugal agitator mixer, sometimes categorized as a planetary mixer.[^104^, ^105^] Centrifugal‐type mixers produce high shear via dual‐axis rotation without the need for agitating blades, which would concentrate energy in one area of the fluid volume. Dual‐axis centrifugation is often performed at speeds from 200 to 3500 rpm, referring to outer‐axis revolutions, while inner axis rotation speed is dependent on the device employed. Some practical advantages of centrifugal mixing include the avoidance of slurry loss and cleaning requirements after each step, in contrast to stirring or milling with foreign implements. However, evidence is scant concerning the optimal range of rotation speeds to ensure homogenization of a slurry without excess expenditure of energy and time in the mixing process.

Due to empirical differences in how each device distributes energy into the target fluid, a given mixing speed applied with one apparatus does not produce the same shear forces if applied with another mixer type. The same obstacle to quantification prevents comparison when mixing larger volumes of slurry, or slurries of greater viscosity, since in these cases proportionally more energy will be required to agitate the slurry to the same effect.^[106]^

Although centrifugal‐type mixers are an efficient means of homogenizing solutions and slurries at higher volumes (50 mL to 1 L) than would be practical using traditional laboratory magnetic stirrers, this method may be challenging when scaled to industrially relevant batches. Commercial lithium‐ion production can be assumed to involve churning large volumes of electrode slurries with shaft‐mounted blades or turbines.^[107]^ For example, a proprietary twin‐screw mixer with integrated injection along a tube was developed for continuous flow slurry mixing.^[108]^ Such automated large‐batch methods cannot be assumed to produce the same results as those used frequently in research publication, such as hand grinding with mortar and pestle.^[109]^ High‐intensity methods such as ultra‐sonication and ball‐milling are also less practical at larger volumes; while the latter may be employed in a pre‐treatment stage, it can also cause undesirable pulverization of active particles.^[110]^

Notwithstanding the efficiency of mixing methods preferred by researchers, cost and practicality may require replacement of these devices in later Na‐ion development with more industrially scalable rotating blade methods. Adopting these processes from lithium‐ion development is complicated by material differences between Na‐ion and Li‐ion, such as discrepancies in surface and structural composition, requiring customization of parameters for each recipe. For example, the current standard anode material for Na‐ion is hard carbon, which contains both graphitic and non‐graphitic carbon.^[111]^ Mixing parameters optimized for graphite particles in Li‐ion studies cannot be directly translated to the more heterogeneous hard carbon, which also differs in surface area and chemistry between different feedstock sources.^[112]^ However, there is greater commonality in the carbon black conductive additives used in both Li‐ion and Na‐ion cells.

Conductive carbon black, generally pyrolyzed from acetylene feedstock,[^113^, ^114^] is the most widely used additive in both cathodes and anodes for both types of batteries. Due to the high surface area (∼64 m^2^/g) of carbon black particles,^[115]^ this component of slurries tends to exert proportionally greater impact on viscosity and surface area through strong inter‐particle associations.^[116]^ This high surface area also promotes carbon black self‐agglomeration, which is associated with decreased cell performance in lithium‐ion cells.^[117]^

Controlled dispersion of carbon black agglomerates was shown to be possible through adjustment of parameters such as mixing time and mixing speed in both cathode^[115]^ and anode slurries,^[118]^ for both non‐aqueous (PVDF) and aqueous carboxymethylcellulose (CMC) systems.^[119]^ Optimized mixing is also necessary to finely control the network of associations between carbon black aggregates, binder globules and active material particles.^[120]^ Faster mixing does not always produce improved performance from slurries, and very high intensity mixing was found to negatively impact the binder‐carbon black network, with consequent decreases in electrochemical capacity.^[121]^ In particular, dry mixing carbon black with cathode active material increased coverage of the larger particles, selectively redistributing the conductive additive on their surface while compromising the electronic connections between them.^[121]^

These results suggest the sequence in which each component is added can have impact on slurry properties as well as the energy applied in mixing. This was observed in Li‐ion electrode tests for both cathodes^[122]^ and anodes,^[123]^ but conclusions from these studies cannot be universally applied to all electrodes. For example, dry ball‐milling of carbon black with active material before adding solvent is considered to improve contact between particles; this improved response in LiFePO_4_ cathodes though not in NMC cathodes.^[124]^ This pre‐milling practice can still be adopted for NMC cathodes by increasing the total proportion of conductive additive,^[125]^ but this displaces active material. Procedures with fewer steps would be more practical for industrial production than adding and blending each component in turn, while results from Li‐ion processing research often recommend more complicated sequencing.^[126]^

Sodium‐ion battery developers should therefore perform a multi‐variable optimization of mixing parameters for any new material in a cathode or anode slurry to maximize an electrode's electrochemical performance. Ample evidence from lithium‐ion research illustrates the importance of not only tuning the ideal ratio of binder,^[127]^ active material and conductive additive,^[128]^ but also the mixing intensity and the order they are added to slurry, which can have a substantial effect on the microstructure and electrochemical properties of an electrode.^[129]^ Even after mixing, slurries were observed to change in viscosity through relaxation of inter‐compositional associations^[130]^ when left to rest before spreading and drying. This temporal variation is another obstacle to repeatability in research settings, where battery production steps are less likely to be automated.

## 3 Spreading and Drying

After mixing, electrode slurries are conventionally spread onto thin metal foils, often called current collectors, and then dried into a solid film. The physico‐chemical properties of the slurry affect how it responds to spreading and drying parameters, with defects at each stage likely to manifest as shortcomings in electrochemical performance.^[131]^ Maximizing repeatability and uniformity of film characteristics is therefore essential to assessing the true potential of applied electrode materials.

### 3.1 Spreading

**Figure 5:**
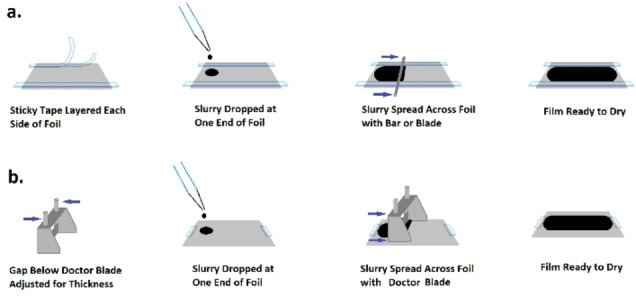
Techniques commonly applied for spreading electrode films at laboratory scale; (a) tape casting and (b) doctor blade coating.

Na‐ion research often requires the production of coin‐sized electrodes in small batches using laboratory bench‐sized equipment, in contrast to wide commercial sheets fabricated by roll‐to‐roll machinery. Testing material mixtures in small volumes is a rational approach to early development, and Na‐ion researchers tend to apply coating methods such as blade or bar spreading to obtain surfaces for testing (see Figure 5). Any visually apparent defects in a film can be selectively avoided when punching out circular samples for coin cell testing. However, the detailed techniques selected when spreading an individual film, often called a “draw down” coating, will affect uniformity across the resulting electrode and repeatability of characteristics between electrodes.

A frequently used device for spreading is a doctor blade, which provides a measured gap through which a slurry can be confined during the spread (Figure 5b). The resulting deposition of slurry will depend on several additional variables, including the speed of blade movement, adhesion to the substrate, and rheological properties.^[132]^ If used in combination with an automatic coating device, blade speed can be more precisely controlled, though effective surface coverage also requires the slurry to adhere to the current collector and resist a tendency to bead. This can be particularly problematic in aqueous slurries using CMC binder, given the high surface tension of water (72 mN m^−1^ at room temperature). Isopropyl alcohol (IPA) can be used to decrease surface tension, and therefore hinder beading to improve adhesion of the wet slurry, although this technique is limited due to the insolubility of CMC in IPA.[^133^, ^134^] This application of additives may be a convenient way to overcome inherent material processing obstacles at the laboratory scale, but it could add cost to the manufacturing process.

The same consideration of viability should apply to achieving substrate cleanliness, which can prevent unwanted side reactions and improve adhesion between film and current collector. Procedures such as chemical rinsing, manual wiping and abrasive buffing^[135]^ are practiced by Li‐ion researchers, all of which could add costly steps to an industrial production process. Since Na‐ion studies generally apply electrode slurries to high purity aluminium, pre‐treatment of the substrate might be unnecessary, therefore these procedures should be used only if and when required to avoid an identified contaminant. Plasma or corona treatment can also be employed to improve adhesion of the slurry to the substrate;^[136]^ it is commonly used in commercial roll‐to‐roll printing as a method to reduce the surface energy of the substrate.

Adhesion at the‐pre‐drying stage could also be addressed by matching coating techniques to slurry characteristics, as described below with evidence from Li‐ion applications. The frequently emphasized ratio of solids to liquid, or solid loading, is only one of several factors influencing the flow characteristics of a slurry.^[137]^ Higher solid loadings can be expected to produce higher viscosity, which presents challenges to uniform spreading. Lower solid loadings contain a higher ratio of solvent, which is a burden to remove at the drying stage. To minimize these disadvantages at research scale, where mixing and spreading is often done manually, a slurry could combine high viscosity with high shear thinning: a rheological property describing decreasing viscosity with increasing shear stress.^[138]^ However, this can present challenges when applied to larger volume methods such as slot‐die coating. Appropriate rheological properties for a spreading technique should be tuned through optimised formulation and selection of binders, since shear thinning behaviour is often dominated by these components of the slurry.^[139]^

An appropriately selected binder will facilitate spreading even at high solid loading, serving as both suspension emulsifier and as adhesive to the dry electrode.^[140]^ Since the percentage of active material in a slurry is a critical factor in electrode performance, binders must perform these functions at very low slurry concentrations (∼2 % of electrode weight^[141]^). This requirement might have contributed to the adoption of the standard binder used in Li‐ion batteries, PVDF, into practice for Na‐ion development despite practical disadvantages such as toxicity of compatible solvents, namely NMP.^[142]^ At industrial scale, the risks associated with PVDF and NMP increase proportionally, motivating fundamental redesign of binders^[143]^ and alternatives to wet slurry coating.^[144]^ Lower‐toxicity solvents such as dimethyl sulfoxide (DMSO)^[145]^ and γ‐valerolactone^[146]^ have been demonstrated with PVDF binders for Li‐ion cathodes but have not been adopted widely. The most frequently used water‐based binder system, a combination of sodium carboxymethylcellulose (CMC) and styrene butadiene rubber (SBR), can also be applied to hard carbon slurries for Na‐ion anodes. While evidence from Li‐ion studies has provided valuable options compatible across chemistries, Na‐ion researchers could also consider novel binders such as sodium alginate demonstrating electrochemical stability, low toxicity and uncomplicated processability with Na‐ion specific electrode active materials.^[147]^

Optimal spreading technique, speed, gap thickness and substrate preparation are therefore dependent on the material makeup of a particular slurry. Pinholes and other superficial defects observable before complete drying of a film can be visual indicators of inefficiencies in matching coating (or mixing) procedures to an electrode formulation.^[148]^ A coating procedure suited to a specific slurry formulation should produce fewer obvious defects such as ridges from non‐uniform thickness, while performance metrics such as adhesion to the current collector can generally only be characterised after electrode drying.

### 3.2 Drying

The evaporation of solvent from the wet slurry causes a coating to begin drying immediately after spreading. This process of evaporation is often accelerated with temperature, airflow, or a combination of the two.^[149]^ At industrial scale, a continuous roll of electrode is dried along a sequence of automated equipment, which can be designed to subject substrate and/or surfaces to multiple zones with separately controlled conditions.^[150]^ Standard laboratory equipment could also be used to select different temperatures for sequential application during the drying process, but most studies tend toward the practice of leaving coatings overnight at a single temperature setting near 100 °C. This convention may be motivated by the availability of vacuum heated exchange compartments on laboratory gloveboxes or the decreased cost and time burden compared with commercial fabrication. However, phenomena including capillary activity and redistribution of suspended particles are directly affected by drying parameters, which consequently impact critical film properties such as adhesion.^[151]^

Extensive evidence from Li‐ion studies have linked faster drying rates to electrode defects such as cracking and poor adhesion to current collector.^[152]^ Though adhesion is not necessarily related linearly to electrochemical performance, electrodes dried at slower rates were found to retain higher capacity than those dried more quickly.^[153]^ Models of physical processes indicate a shrinkage phase, when solvent evaporates uniformly across the wet surface, followed by a longer phase, in which solvent is constrained within columns between settled solid particles. The completion of this later phase could be accelerated by applying higher heat or airflow, but this could still exacerbate the separation of slurry components into layers, which is shown to be a detriment to adhesion.^[154]^

During the drying process, particles of higher density are driven by gravity to settle toward the current collector while lower‐density binder tends to accumulate at the upper surface. This gradient decreases binder concentration at the substrate, where it is most needed to promote adhesion of the film to current collector and to buffer mechanical stress at this interface during electrochemical cycling.^[155]^ Higher drying rates increase this gradient by exacerbating separation forces such as sedimentation and rising viscosity while limiting solvent available for correction by diffusion.^[156]^ Adhesion loss from binder migration leads to delamination of electrodes from current collectors during battery operation, therefore drying rates should be minimized when fabrication time is not restricted. This was shown in both NMP‐based and aqueous slurries, though differences in solvent evaporation and binder chemistry should be considered when selecting drying parameters.[^157^, ^158^]

An additional post‐dry heating step is sometimes applied to ensure thorough evaporation of residual water, which could compromise electrochemical activity even in trace amounts.^[159]^ This applies even to nonaqueous solvent‐based slurries, since water vapour adsorbed onto the film surface after drying would interfere with electrochemical efficiency by reacting with cell electrolyte.^[160]^ As with initial electrode drying, the time and temperature required for this final “baking” process should be determined through validation experiments for each system since advantageous parameters will depend on electrode microstructure.^[161]^ The sequence of drying steps is an additional temporal variable for investigation, and the final heating may be implemented before, during or after additional treatment steps such as compression.

## 4 Electrode Pre‐Treatment

Dried cathode and anode films, usually between 50 μm and 100 μm thick, are sometimes cut and used immediately for experiments such as in coin cells or reusable Swagelok‐type cells. Characteristics of film microstructure can also be tested immediately after drying, including scanning electron microscopy and energy dispersive X‐ray analysis to assess inter‐particle associations and component distribution.^[162]^ Additional post‐drying processing steps have been adopted by Li‐ion producers even in cost‐sensitive settings, based on resulting enhancement of cell performance. Na‐ion researchers should therefore determine for each electrode formulation how processes such as calendering, electrode wetting and SEI formation can be adjusted to provide quantifiable advantages.

### 4.1 Calendering

**Figure 6:**
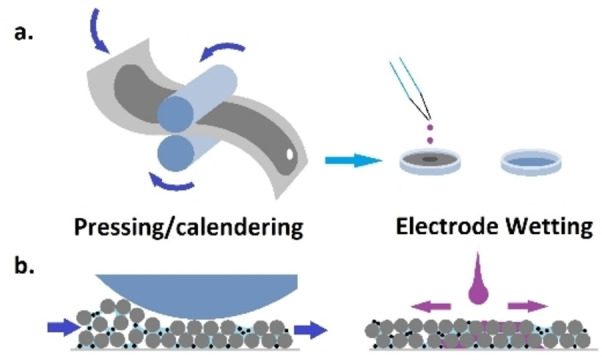
Diagram of electrode pressing and wetting processes, assuming a disc is cut to coin cell size between these steps, shown conceptually from a) lab user and b) cross‐sectional perspectives.

Compression of dried films against current collectors, often called calendering, as shown in Figure 6, is a standard practice in Li‐ion electrode manufacturing to increase energy density and structural homogeneity.^[163]^ The air gaps left by evaporated solvent during the drying stage provide essential voids for electrolyte to fill, but excessive porosity compromises electrode energy density, adhesion and cycling stability.^[164]^ Pressing the film decreases this porosity, while simultaneously increasing interactions with the current collector.^[165]^ The optimal pressure, speed and temperature to set calender rollers depends on physicochemical qualities such as inter‐particle cohesion and elasticity imparted by the binder.^[166]^ These properties vary with both material formulation^[166]^ and earlier processing steps, therefore calendering parameters validated for one electrode type are unlikely to be ideally suited to other systems.

Adding complexity to the selection of these parameters, calendering intensity may be reported in units of gap size,^[167]^ compression force^[168]^ or estimated electrode porosity.^[169]^ Differences in device design, including roller speed and diameter, also affect the pressure exerted by calender equipment on a film.^[169]^ These variables complicate comparison between studies and hinder standardization of calender methods.

Researchers can quantify some of these equipment‐generated variables by separately testing the effect of stack compression.^[170]^ By exerting compression in the perpendicular axis on small film samples, effects of uniformly applied force on porosity and electrode performance can be characterised independent of roller variables such as surface shear. Film thickness and surface microstructure after uniform compression in ideal conditions can subsequently be used to compare the effects of compression with calender rollers.

The adhesion of the film to current collector may also be improved by compression, with direct implications for cell performance.^[155]^ While no standardised test is widely practiced to quantify electrode‐substrate adhesion, custom designed peel‐off techniques are proposed in Li‐ion electrode studies, demonstrating multiple formulation and processing factors linking compression with subsequent adhesion.^[171]^ Additionally, calendering tests on Li‐ion electrodes often apply compressive force with unheated rollers, while the application of a temperature just below the melting point of the selected binder should mitigate film damage caused by roller pressure.^[172]^

Compression force applied to increase energy density and adhesion in Li‐ion electrodes must be selected to balance these advantages with increased risk of fractures severing interparticle connections, which can also be caused by the stress of compression.^[173]^ In contrast, such detailed investigation on the effects of calender pressure on Na‐ion electrodes is under‐reported in literature, with consequences of compression varying from improved cycle life^[174]^ to decreased rate performance^[175]^ for different parameter/material combinations. Each electrode formula should be expected to respond uniquely to calendering, with consequent decreases in porosity, potentially increasing performance while simultaneously complicating the following step of electrode wetting.^[176]^

### 4.2 Wetting

After calendering, dry electrodes are cut to size and shape according to cell dimensions, followed by assembly into a stack. The saturation of cell parts with electrolyte, called wetting, is an essential step to maximize active electrochemical interfaces. Techniques for wetting vary greatly, even between groups experimenting with identical materials and cell sizes. For example, pre‐soaking electrodes and separator with electrolyte before assembly can be helpful in coin cell research, while some groups also advocate electrolyte application to exterior surfaces of current collectors.^[177]^

In contrast, pouch cells filled with electrolyte after assembly may require several “top‐ups” as fluid injected from one side of the cell gradually permeates across the length of electrodes, and a vacuum might also be applied to accelerate the release of gas bubbles lodged in micro pores.^[178]^ Delays to wetting present a cost disadvantage in commercial operations, which has motivated the development of methods for investigating wetting rates.^[179]^ Evidence from these Li‐ion cell studies demonstrates direct performance impacts of under‐wetting and contrasting thresholds for different material‐electrolyte combinations,^[180]^ with implications for Na‐ion research as well.

Radiological or optical monitoring after adding dye to electrolyte can allow visualization of wetting progress through a pouch cell, though any additive must be verified not to change fluid qualities such as viscosity and surface tension.^[181]^ The high viscosity and surface tension of standard carbonate electrolyte solvents in Li‐ion and Na‐ion cells (EC and PC) is a detriment to efficient wetting, motivating the use of low‐viscosity solvents (DMC, EMC) and purpose‐based additives, including surfactants and even water.^[182]^ Elevating temperature during the electrolyte filling process (from 23 °C to 55 °C^[183]^) can also improve wetting rates by decreasing viscosity,^[183]^ presenting less risk of contamination or electrochemical side reactions than introducing additional solvents.

Porosity and pore shape also strongly influence wetting efficiency, therefore electrodes calendered to high density will take longer to wet.[^176^, ^183^] Compression forces during processing can also cause pores deeper in the electrode film to close, resulting in electrochemically inactive voids inaccessible to wetting with electrolyte.^[184]^ An ideal pore alignment perpendicular to the current collector would theoretically prevent such “dead zones” by optimizing wetting and active surface area,^[185]^ but this nanostructuring would require specialized processing with cost implications at commercial scale.^[186]^

Even where production time is not constrained, awareness of cell wetting is a prerequisite to precisely evaluating electrochemical performance. For example, PVDF was found to swell upon electrolyte contact, causing visible deformation such as curling of coin cell electrodes.^[187]^ Wetting surfaces before or during assembly allows observation through visible signs of saturation, and coin cell experimental designers can exploit this capability for control of electrolyte volume and distribution. Pouch cells filled with electrolyte from one side should not be assumed to wet as efficiently as coin cells, since in‐plane wetting progresses more slowly than in the through‐plane direction, and the separator is likely to wet more slowly than electrodes.^[188]^

Separators in Na‐ion research often match standard material used in commercial Li‐ion cells, consisting of a polymer nanofiber matting such as polypyrrole or polyethylene,^[189]^ which may be treated through grafting or irradiation to enhance ionic conductivity.^[190]^ Alternative materials such as glass microfibre^[191]^ are also supplied pre‐made for use as separators in battery research. As a result, Na‐ion battery researchers can select from a range of separator types, ready for use without further processing in the laboratory. In contrast, development of novel separators for Na‐ion cells frequently involves specialised processing equipment such as electrospinning to achieve a desirable nanostructure.[^192^, ^193^] Pores in separators must be sufficiently small and tortuous to obstruct dendritic growth,^[194]^ but this consequently restricts the flow‐through of liquid electrolyte during wetting.

As with electrodes, separator wetting time is dependent on porosity and electrolyte viscosity, and unwetted surfaces will hinder cell electrochemical activity.^[195]^ When researchers select a separator material, wetting efficiency should be considered in addition to mechanical strength and thermal safety. Separator wetting can also be affected by ionic properties of the selected redox‐active salt, independently from electrolyte rheology,^[182]^ which further justifies systematic optimization of wetting for unique cell formulations and dimensions.

One practical method to quantify wetting efficiency is electrochemical impedance spectroscopy (EIS), which could be used either during or after wetting using standard potentiostat equipment.^[196]^ Although EIS is a relatively quick and non‐invasive method to assess wetting of cell micropores, it should be used with caution since even a small input current signal can alter interfacial reaction rates, possibly affecting the SEI formed in a controlled first charge during the last processing step.

### 4.3 Formation

Sodium‐ion cell fabrication steps are not complete after filling with electrolyte and sealing. Until the anode is sodiated, the cell cannot be used as a source of energy. The first cell charge is therefore an essential production process, during which the SEI will begin to form on the anode surface. The SEI will continue to evolve with each subsequent charge throughout operational life, but the characteristics of this layer are strongly influenced by cell conditions during the first cycles. In Li‐ion cells, SEI thickness, composition and electrical resistance are managed by control of critical variables including temperature and charge/discharge current.^[197]^

Na‐ion SEI formation is also likely to be affected by these parameters, such as increased stability of layers formed by cycling at lower C‐rates. However, SEI layers on hard carbon Na‐ion anodes have been observed to be less stable than those on graphite Li‐ion anodes.^[198]^ For this reason, evidence guiding best practice in Li‐ion formation protocols cannot be assumed applicable to Na‐ion SEI formation, which is an under‐explored area of research.[^44^, ^199^]

Further study of Na‐ion formation protocol could potentially improve capacity retention and decrease first‐cycle irreversible capacity loss associated with the SEI, the primary performance barriers for these cells.[^200^, ^201^] Chemical additives such as FEC are often proposed to improve capacity retention through SEI manipulation,[^202^, ^203^] but this should follow fundamental analysis relating initial capacity decreases to each formation parameter, including consideration of subtler aspects such as scan rate^[204]^ and rest periods.^[205]^ These process‐performance links can often be revealed with straightforward single‐parameter tests,[^206^, ^207^] while more detailed chemical characterization methods can be reserved for later studies detailing the mechanistic reasons for observed SEI changes.^[208]^

In a pouch cell, SEI growth is likely to contribute proportionally more to degradation of performance than in a coin cell, which is attributed to weaker compressive forces.^[209]^ Another consequence of upscaling battery size above coin cell is increased accumulation of gaseous reaction products in a sealed cell, primarily during the first charge‐discharge cycles.^[210]^ A technique employed to improve electrolytic performance is to “degass” it; in Li‐ion cells this enables the efficient movement of the lithium ions, thereby improving charging and discharging performance.^[211]^ For example, Xiong, Hynes & Dahn^[212]^ performed degassing of Li‐ion pouch cells by a 2‐step process: by cutting the pouch cell open and then resealing it under vacuum after the cell was charged to 3.5 V at C/20 and held for an hour, this process was repeated after the cell was charged to 4.5 V and held for an hour again. The authors have pointed out that these voltages were determined based on in‐situ measurements during the first charging cycle.^[212]^

Identical processes, albeit with variations, have been considered for Na‐ion cells as well. For a Na‐ion pouch cell comprising of a hard carbon anode and a NaNi_1/3_Fe_1/3_Mn_1/3_O_2_ cathode, degassing was performed after aging for 24 h at 45 °C, cutting and resealing as a single step process.^[213]^ Lee et al.^[214]^ degassed a Na‐ion cell having hard carbon anodes, Na_0.9_Ca_0.035_Cr_0.97_Ti_0.03_O_2_ cathodes and 1 M NaPF_6_ electrolyte during the first charging cycle, in the voltage window of 1.0 to 3.0 V.

Another important role of degassing is to enable the release of excess intra‐cell pressure which may build up later during the cell cycling, which Sathiya et al.^[215]^ have exhibited experimentally. The authors showed that unless degassing is performed, electrode decomposition (the sacrificial carbonate precursor of the layered P2 type cathode, which decomposes at around 4 V versus Na/Na^+^) results in the undesirable build‐up of excess gas and increased intra‐device pressure (by ≈0.05 bar), which negatively influences the cell performance.^[215]^ Furthermore, degassing of the pouch cells prior to cycling resulted in the electrode resistance undergoing a dramatic decrease; Kumar et al.^[216]^ performed degassing inside a glovebox after charging and discharging at C/40, showing that the cells are able to deliver a Coulombic efficiency of ≈90 % and retain about 50 % of original capacity, after 70 cycles of operation.

Employing a different approach, Yu et al.^[217]^ performed the degassing of a Na‐ion full pouch cell having NaTi_2_(PO_4_)_3_/CNF anode and Na_3_V_2_(PO_4_)_3_/CNF cathode by a gradual injection of the electrolyte onto a glass fibre separator, with the authors reporting a maximum capacity output of 126 mAh g^−1^, and an initial coulombic efficiency of 93.6 % which improved to 99 %.^[217]^ These results suggest degassing can play an important role in improving Na‐ion pouch cell performance.

While Na‐ion batteries constructed using different electrode active materials are often compared in literature, contrasting temperature, current and voltage parameters used during cycling for formation prevents like‐for‐like comparison between studies. Since SEI composition varies with electrode and electrolyte formulation, several combinations of cycling parameters and conditions should be tested to optimize performance of a novel active material. Conversion‐type electrode materials also change fundamentally during the first sodiation, which is sometimes performed in a separate step prior to assembly of a full cell for performance testing.[^218^, ^219^] Such pre‐sodiation ensures an abundant supply of sodium in a full cell, at the cost of additional process steps.

In addition to traditional metrics such as half‐cell charge capacity and coulombic efficiency, post‐cycle tests such as EIS can quickly determine interfacial impedance after a defined number of cycles to simplify the task of screening suitable formation parameters for each electrode/electrolyte combination. Since formation is the most cost‐intensive step in commercial Li‐ion battery production,[^220^, ^221^] advances in this process are also likely to be essential for the development of economically competitive Na‐ion batteries.

## 5 Conclusions and Outlook

This text aimed to summarize key underexplored topics in cell production applicable to Na‐ion researchers. As heterogeneous aggregates, Na‐ion cells tend to be constructed in separate parts prior to assembly, compounding multivariate factors even at research laboratory scale. Parameters selected when mixing materials, drying, then pressing and wetting electrodes with electrolyte can impact the mechanical, electrical and chemical characteristics of the resulting cell. Even a minor adjustment in cycle conditions during the final steps, SEI formation and testing, likely influences cell capacity retention due to the sensitivity of intercalating anodic interfaces. Since the outcomes of each step affect the inputs to all following processes, the initial actions of electrode slurry mixing and drying may be the most difficult to optimise through cause‐effect links to performance metrics such as cell energy density and cycle life.

Due to the complex dependencies between interparticle chemistry, rheology and thermodynamics, no single formula can be applied to every combination of slurry composition and procedural variables. However, certain physical characteristics and mechanical responses shared across heterogeneous particle suspensions can be anticipated, of which better understanding should assist in the design of experiments defining process‐structure links. Insight from analysis of Li‐ion cells suggest slurry properties can be tuned with adjustment of agitation speed and sequence of mixing, while pre‐mixing dry components prior to adding solvent can enhance interaction between particles of active material and conductive additives. Accelerated film drying with higher temperature or airflow is likely to exacerbate segregation of binder from the current collector interface, but an understanding of different drying phases combined with investigation into alternative drying protocols could reveal opportunities for increased efficiency with proportionally lower risk of electrode delamination.

In summary, the authors recommend researchers give particular attention to the following processing considerations:

1. When mixing either electrode or electrolyte components, a range of speed and duration should be evaluated with early‐stage characterisation. This may include traditional methods to assess particle fragmentation or post‐drying film microstructure, while metrics such as viscosity and surface tension can be valuable indicators of practicality for larger‐scale production.
2. Repeatability of results across identical cells should be reported, in addition to the performance of an average experimental cell. Conductive characterisation of electrodes after drying, or of electrolyte used at different time points, can be performed quickly using basic two‐ or three‐ point electrochemical methods, such as EIS to monitor interfacial phenomena from wetting to SEI growth. Assessing these metrics prior to long‐term cycling can aid in Na‐ion material‐specific troubleshooting and indicate key sources of inter‐cell variability.
3. C‐rate and voltage windows used in formation and testing should be rationally selected and justified for Na‐ion tests, to build knowledge on the topic. Parameters such as temperature, CC–CV thresholds and inter‐cycle rest periods are important to include in research publication since these can influence the measured capacity and cycle life of novel cells.

While the production variables most critical to Na‐ion cell performance might match those for Li‐ion cells, optimal processing parameters for each material combination can only be determined through validation for each case. Scant reporting of these essential methodological details in research papers limits the applicability of new work to the Na‐ion development community and its relevance to future commercialisation. Enhancing transparency of laboratory‐scale procedures within scientific publications could multiply their informative and translational value while contributing to quality control of practices across the Na‐ion battery research community, consequently benefitting all stakeholders.

## Conflict of interest

The authors declare no conflict of interest.

## Biographical Information

*Dr M. Anne Sawhney is currently a postdoctoral researcher in electrochemical energy storage for SPECIFIC within the Faculty of Science and Engineering at Swansea University. Her focus is the efficient processing, testing and evaluation of hard carbon anodes for sodium‐ion batteries, particularly quantification of electrical/mechanical characteristics to associate manufacturing techniques with consequent impacts on cell performance*.

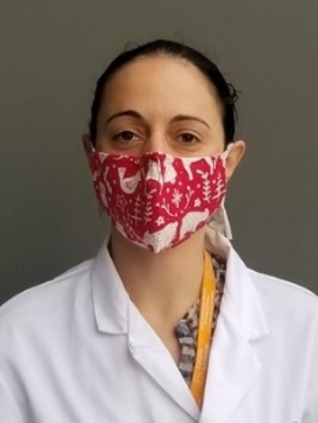

## Biographical Information

*Dr Malik Wahid is currently working as DST INSPIRE Faculty at the Department of Chemistry, National Institute of Technology (NIT) Srinagar, India. Previously, he held the postdoctoral position at the Indian Institute of Science Education and Research (IISER‐Pune) with Professor Ogale. He did his Ph.D. from CSIR‐NCL focusing on the carbon materials for battery and supercapacitor applications. Before this, he received his M.Sc. in Physical Chemistry from Kashmir University (K.U). Dr. Wahid is an Electrochemist and Material Scientist. His current research interests include Electrochemistry of alkali ion systems, Electrocatalytic water splitting, and Electro‐organic Chemistry*.

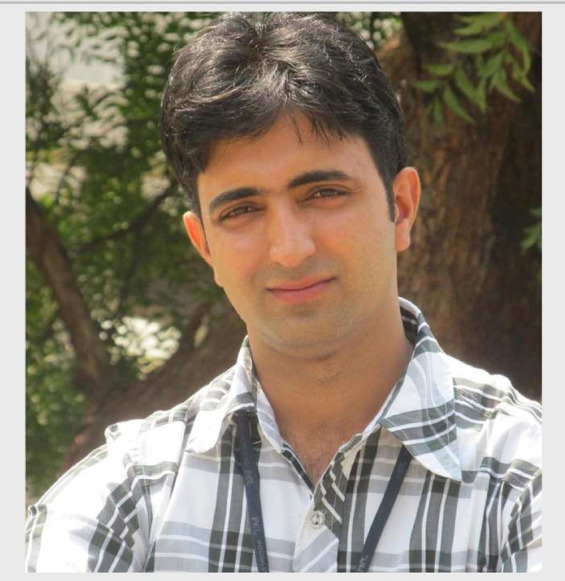

## Biographical Information

*Dr Santanu Muhkerjee is currently a Technology Transfer Fellow – Electrochemical Energy Storage at Swansea University, working on developing innovative sodium ion batteries. He obtained his PhD in 2017 from the University of Louisville (USA) and has subsequently also worked in the US and France. His expertise lies in materials synthesis, characterization and application for electrochemical energy storage*.

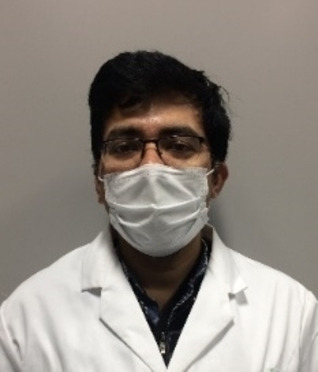

## Biographical Information

*Rebecca Griffin is a second year PhD candidate studying at the University of Swansea, UK. Her passion for sciences started during her BSc Hons and MSc by Research degrees at Lancaster University, UK, in Natural Science with a focus in chemistry. Her current research interests are within the field of energy storage, specialising in solid‐state batteries, electrochemistry and photochemistry*.

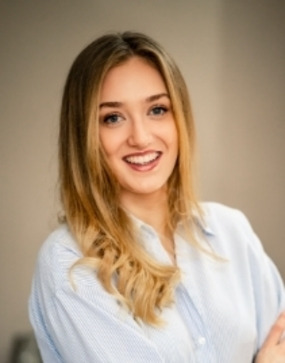

## Biographical Information

*Dr Alexander Roberts is Professor in Energy Storage within the Centre for Clean Growth and Mobility (Coventry University), a Faraday Institution Industrial Fellow (in partnership with Nyobolt Ltd.) and Theme Lead for Energy Storage programmes and research at Coventry University. He has 15 years’ experience engaging in energy storage research, working from materials discovery through to device development, design and testing. He currently holds a range of commercial R&D and consultancy contracts focused on cell development and testing, across a range of companies, including component and cell manufacturers and developers from the UK, Europe and Asia*.

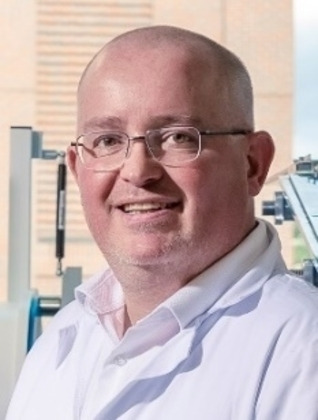

## Biographical Information

*Prof. (Dr) Satishchandra Ogale is currently working as the Director, Research Institute for Sustainable Energy (RISE), The Chatterjee Group's (TCG) Centres for Research and Education in Science and Technology (TCG‐CREST), Kolkata, and also as Emeritus Professor at the Department of Physics and Centre for Energy Science, Indian Institute of Science Education and Research (IISER) Pune. Previously he also held positions at CSIR‐NCL as Chief Scientist (9 years), Visiting Professor and Senior Scientist at University of Maryland, College Park, USA, (10 years) and Faculty and Chair (1992–1995) of the Physics Department at Pune University (16 years)*.

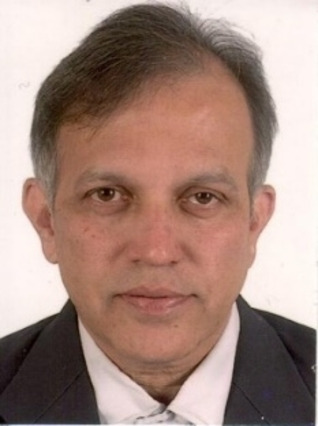

## Biographical Information

*Dr Jenny Baker is a Senior Lecturer Mechanical Engineering at Swansea University and an EPSRC Fellow. Her group focuses on sustainable manufacturing of sodium ion batteries and life cycle assessment of energy storage ss part of the Sustainable Product Engineering for Innovate Functional Industrial Coatings (SPECIFIC) project at Swansea University. Her expertise lies in manufacturing within a circular economy with 13 years′ experience in aerospace manufacturing prior to her academic career*.

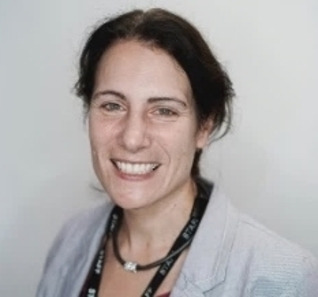
